# Optical genome mapping with whole genome sequencing identifies complex chromosomal structural variations in acute leukemia

**DOI:** 10.3389/fgene.2025.1496847

**Published:** 2025-04-02

**Authors:** Meng-Ju Melody Tsai, Hsiao-Jung Kao, Hsiao-Huei Chen, Chih-Hsiang Yu, Yin-Hsiu Chien, Wuh-Liang Hwu, Pui-Yan Kwok, Ni-Chung Lee, Yung-Li Yang

**Affiliations:** ^1^ Department of Pediatrics, National Taiwan University Hospital and College of Medicine, National Taiwan University, Taipei, Taiwan; ^2^ Department of Pediatrics, National Taiwan University Hospital Yunlin Branch, Yunlin, Taiwan; ^3^ Institute of Biomedical Sciences, Academia Sinica, Taipei, Taiwan; ^4^ Institute of Statistical Science Academia Sinica, Taipei, Taiwan; ^5^ Department of Medical Genetics, National Taiwan University Hospital, Taipei, Taiwan; ^6^ Center for Precision Medicine, China Medical University Hospital, China Medical University, Taichung, Taiwan; ^7^ Cardiovascular Research Institute, Institute for Human Genetics, University of California, San Francisco, San Francisco, CA, United States; ^8^ Department of Dermatology, University of California, San Francisco, San Francisco, CA, United States; ^9^ Department of Laboratory Medicine, National Taiwan University Hospital, and Department of Laboratory Medicine, College of Medicine, National Taiwan University, Taipei, Taiwan; ^10^ Department of Laboratory Medicine, National Taiwan University Cancer Center, Taipei, Taiwan

**Keywords:** chromosomal structural variation, optical genome mapping, whole genome sequencing, Leukemia, Bionano

## Abstract

**Introduction:**

Chromosomal structural variations (SVs) play an important role in the formation of human cancers, including leukemias. However, many complex SVs cannot be identified by conventional tools, including karyotyping, fluorescence *in situ* hybridization, microarrays, and multiplex ligation-dependent probe amplification (MLPA).

**Methods:**

Optical genome mapping (OGM) and whole genome sequencing (WGS) were employed to analyze five leukemia samples with SVs detected by karyotyping, MLPA, and RNA sequencing (RNA-seq). OGM was performed using the Saphyr chip on a Bionano Saphyr system. Copy number variation and rare variant assembly analyses were performed with Bionano software v3.7. WGS was analyzed by the Manta program for SVs.

**Results:**

The leukemia samples had an average of 477 insertions, 457 deletions, and 32 inversions, which were significantly greater than those of the normal blood samples (*p* = 0.016, 0.028, and 0.028, respectively). In Case 1, OGM detected a sequential translocation between chromosomes 5, 8, 12, and 21 and *ETV6::RUNX1* and *BCAT1::BAALC* gene fusions. Case 2 had two pathogenic SVs and a *BCR::ABL1* fusion. Case 3 had one pathogenic SV and an *IGH::DUSP22* fusion. Case 4 had two pathogenic SVs and a *CBFB::MYH11* fusion. Case 5 had an *STIL::TAL1* fusion. All breakpoint sequences were defined by WGS. An *IGH::DUX4* fusion previously found by RNA-seq in Case 3 was not confirmed because *DUX4*, which has multiple pseudogenes, was refractory to OGM and WGS analyses.

**Conclusion:**

OGM is a fundamental tool that complements G-banding analysis in identifying complex SVs in leukemia samples, and WGS effectively closes the gaps in OGM mapping.

## 1 Introduction

Chromosomal structural variations (SVs) are defined as regions of DNA larger than 1 kb that show changes in copy number (deletions and duplications), orientation (inversions), or chromosomal location (insertions and translocations) between individuals (Escaramis et al., 2015). SVs can affect gene expression and are associated with a wide range of genetic and cancer-related conditions. Multiple tools, including karyotyping, fluorescence *in situ* hybridization (FISH), microarrays, multiplex ligation-dependent probe amplification (MLPA), PCR (polymerase chain reaction) and RT-PCR (reverse transcription polymerase chain reaction), have been used to analyze SVs (Smeets, 2004; Vissers et al., 2005). Karyotyping has a maximum banding resolution of approximately 5 Mb (Neveling et al., 2021). FISH requires *a priori* knowledge of the loci and has limited throughput (Neveling et al., 2021). Microarrays have a resolution of a few kb but are unable to detect balanced chromosomal aberrations, including translocations and inversions (Neveling et al., 2021). In addition, microarrays are limited in their ability to detect low-percentage clones or subclones, particularly in cancer cells. These limitations of conventional SV analytic tools cause difficulties in the analysis of leukemia samples, which frequently exhibit complex SVs. Acute leukemias, including acute myeloid leukemia and acute lymphoblastic leukemia, are hematologic malignancies originating from progenitor cells that have acquired chromosomal aberrations or somatic mutations that provide selective advantages. SVs play a pivotal role in the pathogenesis of leukemia, and chromosomal aberrations are detected in up to 65% of adult acute leukemia patients and 75% of pediatric patients (Mrozek et al., 2004). Knowledge of chromosomal aberrations plays an essential role in defining the etiology of leukemias, establishing risk and prognosis, and guiding therapeutic strategies (Alaggio et al., 2022; Khoury et al., 2022; Dohner et al., 2022). The workflow for diagnosing acute leukemia typically entails a battery of tests, including karyotype analysis, FISH studies targeting common chromosomal deletions and translocations, and reverse transcriptase PCR (RT–PCR) or RNA sequencing (RNA-seq) (Baranger et al., 2016). Although these tests are time-consuming, a significant portion of complex SVs still cannot be identified, and the breaking point sequences are frequently unknown.

Short-read next-generation sequencing (NGS), including whole exome sequencing (WES) and whole genome sequencing (WGS), is commonly used to detect sequence variations but has a low sensitivity for detecting SVs. Long-read sequencing methods, such as PacBio or Nanopore sequencing, are more capable of identifying SVs, but the costs are high (Marx, 2023). Bionano optical genome mapping (OGM), a cutting-edge technology for analyzing ultrahigh-molecular-weight DNA molecules, can provide high-resolution and long-range genome-wide assessments of structural anomalies (Sahajpal et al., 2021; Dremsek et al., 2021). In OGM, DNA is typically fluorescently labeled through covalent modification at CTTAAG hexamer motifs, resulting in genome-wide labeling of approximately 14–17 signals per 100 kb in sequence-specific patterns. The labeled DNA is loaded onto silicon chips with hundreds of thousands of parallel nanochannels, where individual DNA molecules are linearized, imaged, and digitized. This imaging technology evaluates the fluorescent labeling pattern of individual DNA molecules to conduct an unbiased assessment of genome-wide structural variants as small as 500 base pairs in size. OGM technology has advanced our understanding of the human genome and improved the diagnosis and treatment of genetic and cancer-related disorders. OGM has the potential to enhance structural variation characterization in hematologic malignancies, especially for complex variants. Integration of OGM into a unified testing pipeline reduces personnel and overall costs for laboratories, facilitating in-depth genomic analysis of rare malignancies. This technology is expected to reveal previously unknown genetic alterations in both common and rare hematologic malignancies, advancing our understanding of disease mechanisms (Smith et al., 2022).

In the present study, we employed OGM to evaluate five leukemia samples known to harbor multiple chromosomal SVs. We found that OGM, as a single test, detected more SVs in these samples than multiple conventional tools and was a very powerful tool for identifying complex SVs. We further demonstrated that WGS alone has low sensitivity for detecting SVs (Nakagawa and Fujita, 2018) and that the sequence gaps left by OGM are easily closed.

## 2 Methods

### 2.1 Leukemia and control samples, and conventional techniques used

Bone marrow aspiration samples were obtained from patients with leukemia. The samples were frozen in 10% dimethyl sulfoxide (DMSO) and 90% fetal calf serum in liquid nitrogen (Yu et al., 2022). SVs were detected in these samples using the following tools. Karyotyping was performed by standard G-banding methods. MLPA kits P036, P327 and P335 (MRC-Holland, Amsterdam, Netherlands) were used for B-cell leukemia samples: P036 for subtelomeric regions; P327 for the ERG gene and intrachromosomal amplification of chromosome 21; and P335 for the *EBF1*, *IKZF1*, *CDKN2A*, *CDKN2B*, *PAX5*, *ETV6*, *RB1* and *BTG1* genes. MLPA kits P383 (MRC-Holland, Amsterdam, Netherlands) was used for T-cell leukemia sample for *STIL::TAL1*, *LEF1*, *CASP8AP2*, *MYB*, *EZH2*, *MLLT3*, *MTAP*, *CDKN2A/B*, *NUP214::ABL1*, *PTEN*, *LMO1*, *LMO2*, *NF1*, *SUZ12*, *PTPN2* and *PHF6*. Gene fusion events were detected by either targeted RT‒PCR or RNA-seq. Peripheral blood samples from five healthy donors were used as controls. The study received approval from the Institutional Review Board of National Taiwan University Hospital.

### 2.2 Sample preparation for OGM and WGS

In this study, peripheral blood cells were used as controls, while bone marrow cells were utilized for leukemia samples. The selection of sample types was guided by the clinical relevance of these sources: peripheral blood cells (n = 5) provide a non-invasive and readily available source for controls, while bone marrow cells (n = 5) are the standard diagnostic sample for leukemia due to their higher yield of leukemic cells.

Ultrahigh molecular weight DNA was extracted from 1.5 million cells. Peripheral blood samples were used within 48 h of collection, and after red blood cell (RBC) removal (RBC lysis buffer, Qiagen), DNA was extracted with a Bionano Prep™ kit (Bionano Genomics). Frozen bone marrow samples were thawed in a 37 °C water bath and washed three times with a 10% DMSO solution, after which DNA was extracted with a Bionano Prep SP Frozen Human Blood DNA Isolation kit (Bionano Genomics). Extracted DNA was quantitated using a Qubit fluorometer.

### 2.3 Optical genome mapping (OGM)

DNA DL-green fluorophore labeling was performed using the Bionano Direct Label and Stain kit. After washing out excess fluorophores, the labeled DNA was loaded on a Saphyr Chip^®^ and analyzed on a Bionano Saphyr system (Bionano Genomics). Optical images of the labeled molecules were used to generate rare variant pipeline assembled genome maps with the default setting of the Bionano Solve pipeline (Bionano software v3.7). All SVs (hg38), including deletions, insertions, inversions, and translocations, were annotated with the Variant Annotation Pipeline.

### 2.4 Whole genome sequencing (WGS)

WGS was conducted on an Illumina NovaSeq 6000 system with an average coverage depth of 30X. The raw sequencing reads were aligned to the hg38 reference genome using the BWA-GATK-ANNOVAR pipeline. SVs identified by the Manta program were searched for in the gaps left by OGM alignments.

### 2.5 Statistics

The statistical analyses were performed using SPSS software (version 25.0 and 22.0; IBM Corp., Armonk, NY, United States). Variables related to leukemia group and the control group were analyzed using the Mann‒Whitney U test for comparison. A p value <0.05 was considered to indicate statistical significance.

## 3 Results

### 3.1 SVs detected by OGM in leukemia and control samples

OGM analysis of the five leukemia samples achieved an effective coverage of >300x in all samples, with an average label density of 15.07 per 100 kb (SD 0.93) and a mapping rate of 87.8% (SD 4.49). After filtering, an average of 1,044 SVs were identified in each sample, including 477 insertions, 457 deletions, 32 inversions, and 73 duplications (Supplementary Table S1). In comparison, an average of 650 SVs were identified in the control samples, including 315 insertions (*p* = 0.016), 284 deletions (*p* = 0.028), 32 inversions (*p* = 0.028), and 17 duplications (Supplementary Table S1). Chromosomal translocation (intertranslocation) was observed only in leukemia samples.

### 3.2 Comparison between OGM and conventional diagnostic tool

OGM detected all previously known SVs except for the *IGH::DUX4* fusion detected by RNA-seq in Case 3. OGM also detected additional SVs in these samples (Table 1).

**TABLE 1:** Structural variations detected by various methods in five leukemia samples.

		Conventional tool			Bionano OGM	
No	Type	Karyotype	Fusion gene	MLPA for specific region	Karyotype generated from OGM (>5 Mb)	Involved gene
1	B-ALL	46,XX,t(5;8)(q31;q24), ?der(12)t(11;12)(q13;p11), idic(21)(p11)[8]/46,XX[12]	ETV6::RUNX1 (RT‒PCR)	Duplication: 21q11.2-22.12 Deletion: ETV6 exon 8 Duplication: ETV6 exons 1–5	46,XX,t(1;2)(q42.3;p25.3), der(5)t(5;8;12;21)(q23;q22.3;p12.1;q22), der(8)t(5;8;12;21)(q23;q22.3;p12.1;q22), der(12)t(5;8;12;21)(q23;q22.3;p12.1;q22), del(12)(p12.1p13.2) der(21;21)(12pter → 12p13.2::21q22 → 21p11.1::21p11.1→21q22::12p13.2→12pter)	ETV6::RUNX1 BCAT1::BAALC
2	B-ALL	46,XX[20]	BCR::ABL1 (RT‒PCR)	Deletion: IKZF1 exons 2–7 Deletion: CDKN2A/2B Deletion: PAX5 exons 7–10	46,XX,t(9;22)(q34;q11), del(9)(p13.3p21.3)	BCR::ABL1 CDKN2A/2B deletion PAX5 exon 7–10 deletion
3	B-ALL	46,XY[20]	IGH::DUX4 (RNA-seq)	No change	46,XY,t(6,14)(p25.2,q32.3)	IGH::DUSP22
4	AML	46,XX,inv(16)(p13q22)[20]	CBFB::MYH11 (RT‒PCR)	Not done	46,XX,inv(16)(p13q22)	CBFB::MYH11
5	T-ALL	46,XY[20]	STIL::TAL1 (RT‒PCR)	Deletion: CDKN2A/2B Deletion: STIL exons 6–12	46,XY	STIL::TAL1 CDKN2A/2B deletion

**FIGURE 1:**
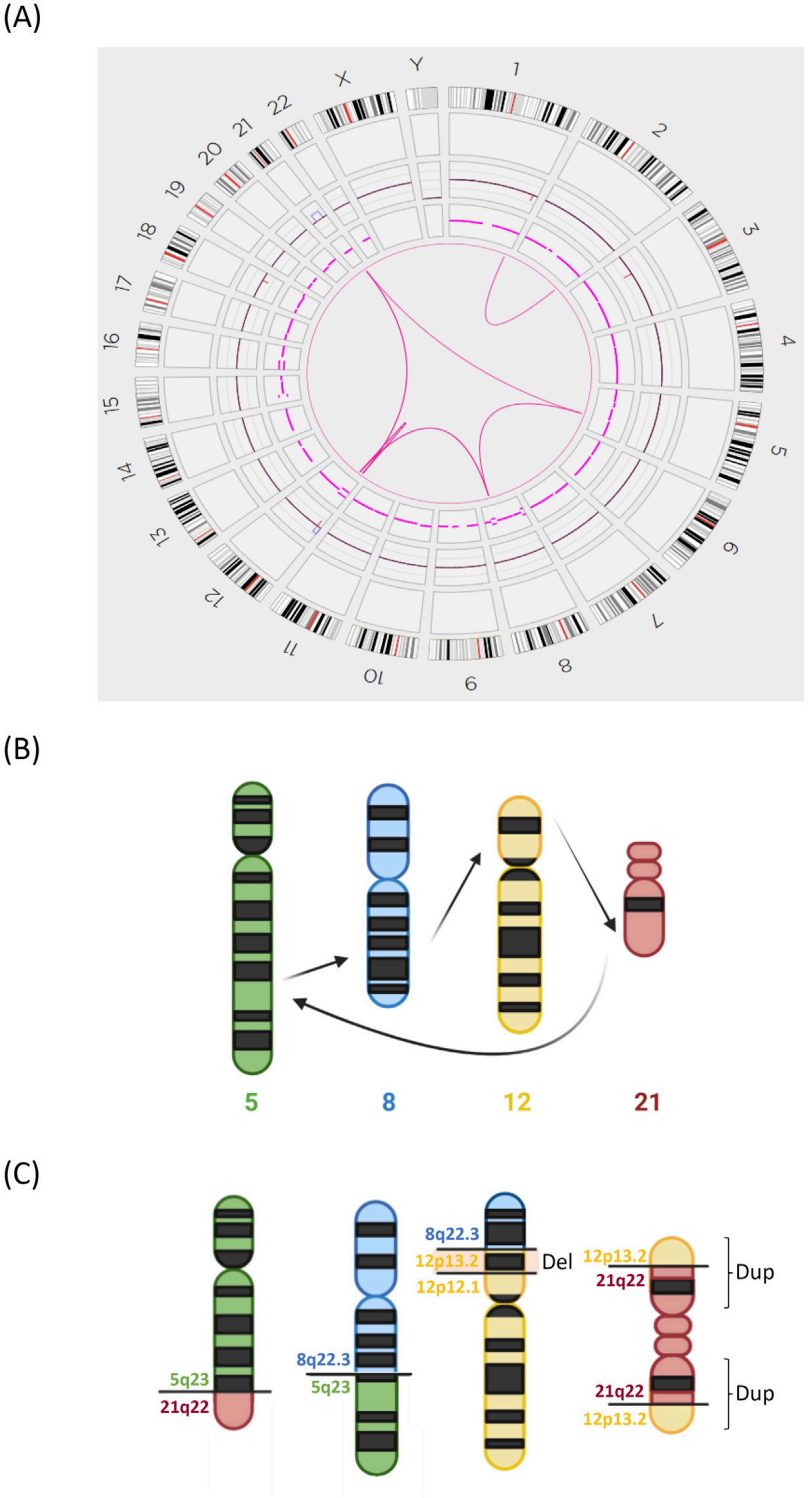
Complex chromosomal translocations identified by OGM in Case 1. **(A)** Circos plot of the interchromosomal translocations detected by OGM. **(B)** Model diagrams illustrating the sequential translocations of chromosomes 5, 8, 12, and 21. **(C)** The structures of the derived chromosomes 5, 8, 12, and 21. There was an additional deletion of chromosome 12p. The derived chromosome 21 was duplicated into an isodicentric chromosome.

**FIGURE 2:**
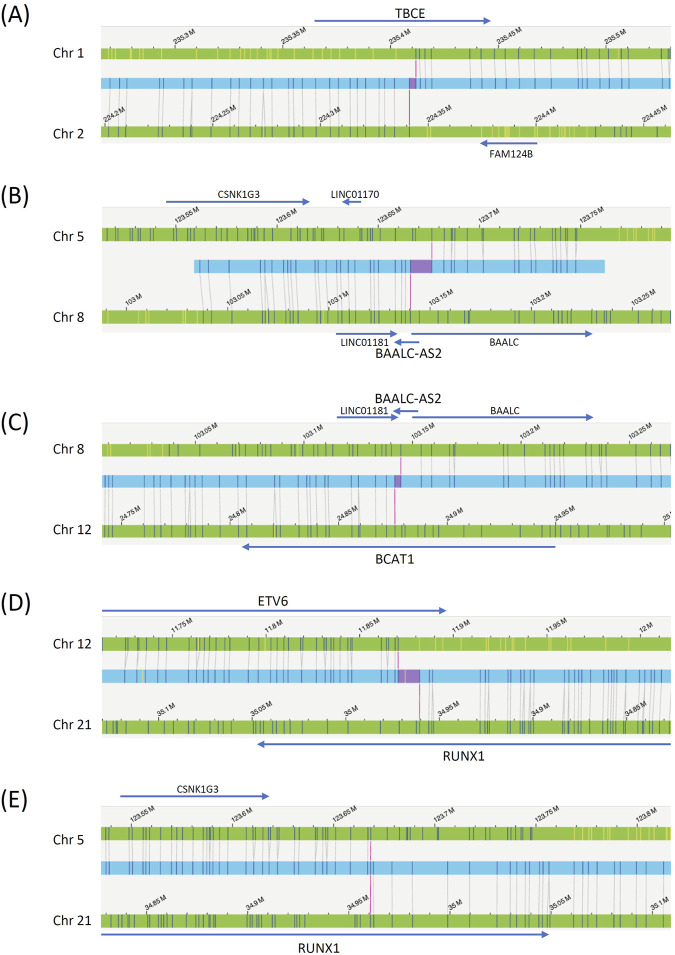
OGM reads determining chromosomal translocations in Case 1. The blue bars are the OGM reads, the green bars are the reference chromosomes, and the gray lines are the mapped OGM probes. Genes surrounding the breakpoints are also labeled. The red bars within the blue bars indicate gaps left by OGM. **(A)** Chromosome 1 to 2 translocation. **(B)** Chromosome 5 to 8 translocation. **(C)** Chromosome 8 to 12 translocation. **(D)** Chromosome 12 to 21 translocation. **(E)** Chromosome 21 to 5 translocation.

Case 1 Conventional tests yielded a karyotype of 46,XX,t(5;8)(q31;q24),?der(12)t(11;12)(q13;p11),idic(21)(p11)[8]/46,XX[12]. The *ETV6::RUNX1* fusion (t(12;21)(p13;q22) (exon 5-exon 3)) was detected by RT‒PCR, and the 21q11.2-22.12 duplication, *ETV6* exon 8 deletion, and *ETV6* exons 1–5 duplication were detected by MLPA. OGM analysis revealed a more complicated karyotype, including sequential translocations between chromosomes 5, 8, 12, and 21 at 5q23, 8q22.3, 12p12.1, and 21q22 (Figures 1, 2). Additionally, a deletion in chromosome 12 (p12.1 to p13.2) was detected only by OGM. The changes in chromosome 21 are more complicated than those observed by conventional karyotyping; for example, 12p13.2 was first translocated to 21q22, and 21q22 was subsequently translocated to 5q23; second, the derived 21q was duplicated, which led to the formation of a dicentric chromosome 21. OGM also detected a translocation between the termini of chromosomes 1 and 2. All gaps at breakpoints mapped by OGM were closed by WGS (Supplementary Table S2), including the *BCAT1::BAALC* fusion between chromosomes 8 and 12.

Case 2 Conventional tests yielded a karyotype of 46,XX in 20 cells, BCR::ABL1 fusion (t(9;22)(q34;q11) (exon 1-exon 2)) by RT‒PCR and deletions of *IKZF1* exons 2–7 (7p12.2), *CDKN2A/2B* (9p21.3), and *PAX5* exons 7–10 (9p13.2) by MPLA. OGM revealed a translocation between chromosomes 9 and 22, which explained the *BCR::ABL1* fusion. OGM revealed a large deletion of chromosome 9p (p13.3 to p21.3), which encompassed 16,321,476 base pairs and contained 245 genes, including *CDKN2A/2B* and *PAX5*. OGM further revealed a smaller deletion of 88,550 base pairs on chromosome 7 involving *IKZF1*. The large deletion (>16 Mb) identified by OGM may have been missed by karyotyping due to the mosaic nature of the cancer cells (with a deletion heterozygosity of approximately 20%) or their reduced proliferative capacity in standard culture conditions. WGS defined the breakpoint for the Philadelphia chromosome as chr9:130813896 fused with chr22:23227330 and chr22:23227334 fused with chr9:130813908.

**FIGURE 3:**
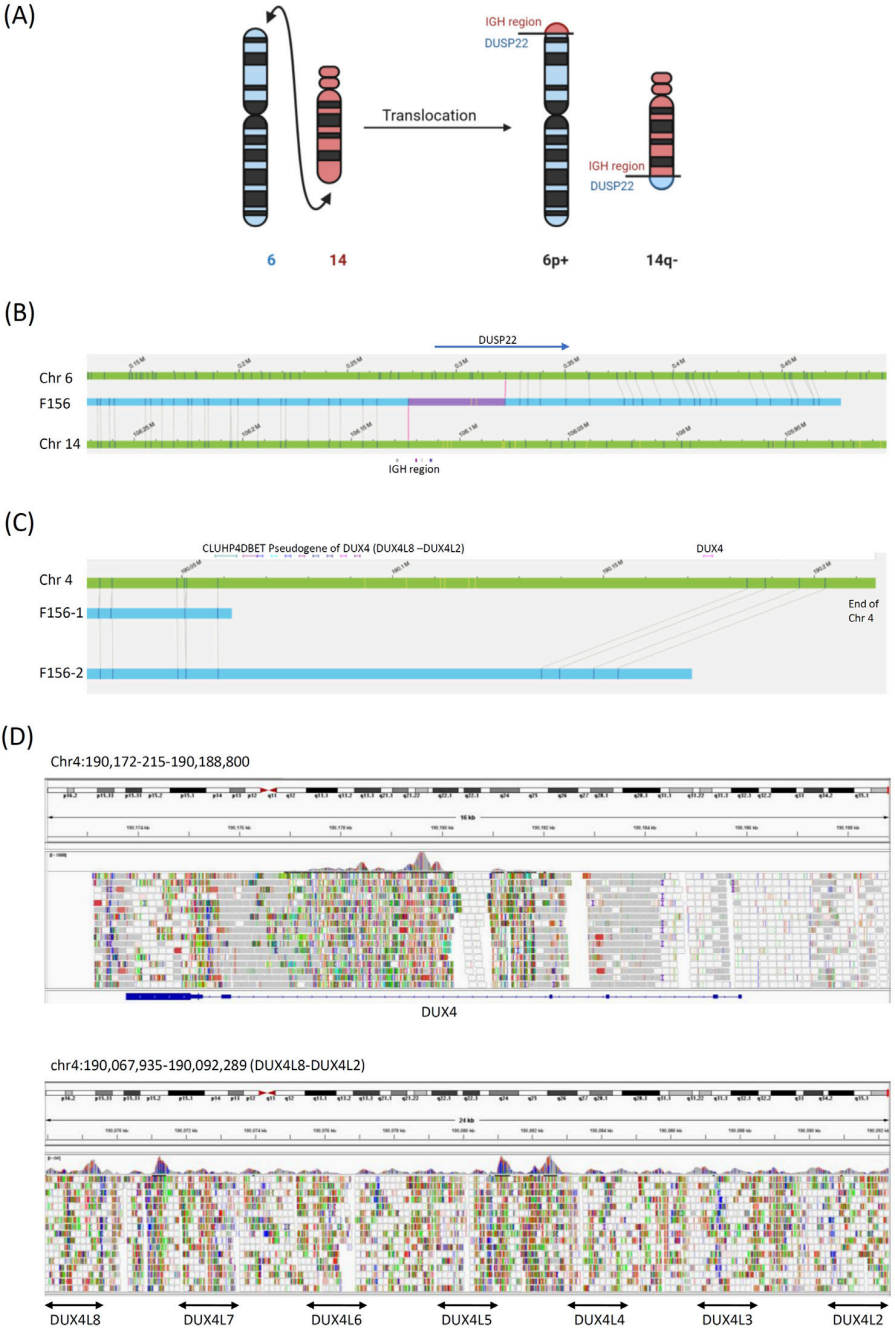
Structural variant in Case 3. **(A)** Model diagrams illustrating the chromosome 6 to 14 translocation. **(B)** OGM reads identifying the chromosome 6 to 14 translocation. The blue bars are the OGM reads, the green bars are the reference chromosomes, and the gray lines are the mapped OGM probes. Genes surrounding the breakpoints are also labeled. The red bars within the blue bars indicate gaps left by OGM. **(C)** The end of chromosome 4, which contains the DUX4 gene, was poorly mapped by OGM because of the lack of probes in this region. **(D)** WGS could not be used to map the DUX gene because of poor alignment due to the presence of multiple DUX4 pseudogenes.

Case 3 The only previously known abnormality in this patient was the *IGH::DUX4* fusion with the highly expressive of *DUX4* gene detected by RNA-seq (Yu et al., 2022). OGM revealed a terminal translocation between chromosomes 6p25.2 and 14q32.3 and an *IGH::DUSP22* fusion (Figures 3A,B). This gene fusion has been associated with chronic myeloid leukemia and lymphoma. (Savage and Slack, 2023; Pedersen et al., 2017) We could not confirm the fusion of *IGH::DUX4*. There were few OGM probes in this region, and WGS could not map this region because of the presence of multiple *DUX4* pseudogenes (Figures 3C,D).

Case 4 Conventional tests yielded a karyotype of 46,XX,inv(16)(p13q22), and the *CBFB::MYH11* fusion (chr16:15721182-67089716) was detected by RT-PCR. OGM/WGS identified both the breakpoint of the *MYH11::CBFB* fusion and 676 genes in the inversion region. Since Case 4 is AML, and prognostic factors for AML are typically SNVs, MLPA was not performed.

Case 5 Conventional karyotyping results were normal, an *STIL::TAL1* fusion was detected by RT‒PCR, and *CDKN2A/2B* (9p21.3) and *STIL* (1p33) deletions were detected by MPLA. OGM determined that the *STIL::TAL1* fusion on chromosome 1 was caused by an 81,839 bp deletion, and another 115,539 bp deletion was detected on chromosome 9, which involved *CDKN2A/2B*.

## 4 Discussion

### 4.1 SVs detected by OGM in leukemia and control samples

In our study, we found that the leukemia samples had an average of 477 insertions, 457 deletions, and 32 inversions, which were significantly greater than those observed in the normal blood samples (*p* = 0.016, 0.028, and 0.028, respectively). Fresh peripheral blood was used as controls, while frozen bone marrow was used for patients. Although freeze-thaw may cause random DNA breakage, the accuracy of OGM was not affected because DNA fragment lengths were sufficiently long in all samples.

### 4.2 Conventional approaches for identifying SVs in leukemia sample

The current study demonstrated the power of OGM as a single test for detecting SVs in leukemia patients. Cytogenetic studies have been shown to be crucial for identifying genes implicated in the development of human leukemia (Nowell, 1993). However, only a portion of gene fusion events can be detected by karyotyping alone. FISH can detect a panel of chromosomal aberrations but requires time-consuming techniques (Neveling et al., 2021). Microarrays are an efficient alternative to karyotyping in leukemia diagnosis, which is mainly associated with SVs (Neveling et al., 2021). Therefore, the most common practice is to design a panel for RT‒PCR or MLPA to detect known SVs involved in leukemia (Smeets, 2004; Vissers et al., 2005). In contrast, OGM efficiently detected SVs in a straightforward way. It would be useful since high hyperdiploid is the most common subtype of leukemia. The cost of OGM is probably similar to the combined cost of conventional tests but less than that of long-read sequencing.

### 4.3 New information derived from OGM/WGS in the present study

In Case 1, OGM detected the *BCAT1::BAALC* fusion. Although the *BCAT1::BAALC* fusion has not been previously reported, both *BCAT1* and *BAALC* are associated with chronic myeloid leukemia (Hattori et al., 2017). BCAT1 catalyzes the initial step of branched-chain amino acid catabolism and is known to promote cancer proliferation and invasion through the activation of either the phosphatidylinositol 3-kinase/protein kinase B/mammalian target of rapamycin pathway or Wnt/β-catenin signal transduction (Nong et al., 2022). *BAALC* expression has also been associated with acute lymphoblastic leukemia and acute myeloid leukemia (Tanner et al., 2001). In Case 3, OGM detected the *IGH::DUSP22* fusion. *DUSP22* is a tumor suppressor gene, and rearrangements in this gene have been associated with favorable outcomes in patients with kinase-negative anaplastic large cell lymphoma (Pedersen et al., 2017). *DUSP22* is located at the end of chromosome 6p, a location difficult to identify by karyotyping. The translocation of the IGH proto-oncogene is a common driver event in leukemia (Tian et al., 2019). The significance of these findings is further underscored by recent observations linking monoallelic 6p25.3 rearrangements with *DUSP22* to lymphoma and leukemia (Savage and Slack, 2023; Arruga et al., 2017). The multiplex reciprocal translocation in Case 1 is intriguing. This variant likely involved sequential translocations of chromosomes 5, 8, 12, and 21. The four chromosomes might be organized together in the nucleus, so the four translocations could occur simultaneously. The use of OGM to detect new fusion genes can provide invaluable insights for cancer research.

OGM detected all SVs known from conventional tools in Case 2, Case 4, and Case 5. OGM also revealed other SVs currently lack established clinical significance, but these information could be valuable for future researches. The clinical implications of these results obtained by OGM are multifaceted. First, OGM offers higher resolution for SVs than by conventional tools like karyotyping or FISH. This leads to improved diagnostic accuracy, which is crucial for personalized medicine. Second, OGM enables the identification of novel or rare SVs, enabling understanding of disease mechanisms or finding new biomarkers useful for classification or prognosis. Third, OGM generates comprehensive SV information that can be generated by multiple conventional tests. Therefore, OGM simplifies diagnostic workflows, reduces turnaround times, and may lower overall costs, making it a powerful tool for advancing precision medicine.

### 4.4 Coupling OGM with WGS

OGM can detect chromosomal translocations, deletions, duplications, and inversions throughout the genome. However, because OGM depends on the location of fluorescence markers within the genome, the exact sequences at the breakpoints have not been resolved. For novel gene fusion events or fusion events in which the reading frames of the downstream gene are important, it may be important to sequence the breakpoint. This can be accomplished by designing breakpoint PCR and Sanger sequencing methods. In the present study, we employed short-read WGS to close the gap in the breakpoint left by OGM. Short-read WGS is thought to have low sensitivity for SVs, and most tools usually generate tens of thousands of SVs from a single analysis; these SVs are almost impossible to interpret (Nakagawa and Fujita, 2018). However, in the present study, when we had already located the breakpoint within a few kb of sequences by OGM, it was quite easy to identify the breakpoint read by WGS and precisely define the breakpoint, including the read frames. Because WGS is inexpensive and quick, it is not a burden to add WGS to OGM, and the entire process can be completed in days. Therefore, short-read WGS effectively close the gap at the breakpoints of SVs detected by OGM. However, short-read WGS itself, or conventional WGS, does not unambiguously detect SVs. A key advantage of OGM/WGS is their ability to bypass the need for cell culture. Karyotyping and FISH typically require 14 days to 1 month for cell culture. While MLPA and RT-PCR are fast, they only target specific regions (Supplementary Table S3). In recent years, artificial intelligence (AI) has aided both conventional cytogenetics and new technologies like OGM to improve cancer cytogenetic analysis (Alain, 2024).

### 4.5 Limitations

In Case 3, the *IGH::DUX4* fusion previously detected by RNA-seq could not be confirmed by either OGM or WGS. *DUX4* is located at the terminal end of chromosome 4 and contains 11 to more than 100 D4Z4 repeats (Dib et al., 2019). Unfortunately, OGM coverage in this particular region was low, and this region was also refractory to short-read sequencing alignment. Recently, long-read sequencing has been shown to detect D4Z4 repeat contraction in patients with facioscapulohumeral muscular dystrophy (FSHD) (Pearson, 2010; Yeetong et al., 2023). Therefore, long-read sequencing may be needed to confirm the *IGH::DUX4* fusion.

## 5 Conclusion

The OGM is a fundamental tool complementing the G-banding analysis, providing the characterization of the chromosomal rearrangement as well as the genes involved, undoubtedly leading to an advance in the knowledge of the biology of the disease and with application in precision medicine.

## Data Availability

The datasets presented in this article are not readily available due to local ethical regulation. Requests to access the datasets should be directed to the corresponding author.
