# Supplementary material for: Optical genome mapping with whole genome sequencing identifies complex chromosomal structural variations in acute leukemia
Source: Front Genet. 2025 Apr 2;16:1496847. doi: 10.3389/fgene.2025.1496847 (PMC12000080; doi:10.3389/fgene.2025.1496847)
Supplement: Supplementary file 1 [file Table1.docx]

Supplementary Table 1. The demographic information of the cases and comparison of the numbers of detected structural variations (SVs) between leukemia and normal blood samples.

|  | Leukemia (n=5) | Control (n=5) | *p* value |
| --- | --- | --- | --- |
| Age (year) | 38 [7-67] | 42 [38-48] | 0.600 |
| Sex (F:M) | 1:4 | 2:3 | - |
| Leukemia type (B:T) | 3:2 | - | - |
| Type of SV |  |  |  |
| Insertion | 447 [343-593] | 315 [253-398] | 0.016* |
| Deletion | 457 [372-550] | 284 [215-401] | 0.028* |
| Inversion | 32 [20-40] | 17 [9-26] | 0.028* |
| Duplication | 73 [28-174] | 27 [7-58] | 0.117 |
| Intrafusion | 1 [0-2] | 1 [0-3] | 0.408 |
| Intertranslocation | 2 [0-6] | 0 [0-0] | 0.054 |
| CNV gain segment | 1 [0-2] | 0 [0-0] | 0.136 |
| CNV loss segment | 2 [0-5] | 7 [0-26] | 0.432 |
| Aneuploidy gain | 0 [0-0] | 0 [0-0] | - |
| Aneuploidy loss | 0 [0-0] | 0 [0-0] | - |

CNV: Copy number variation. The numerical values are presented as average [range].

Supplementary Table 2. Breakpoints and disrupted genes of five leukemia samples

| No | Type of change | Breakpoint position | Disrupted gene |
| --- | --- | --- | --- |
| 1 | Translocation | 5:123669198  21:34961755 | Intergenic region  RUNX1 |
|  | Translocation | 5:123669242  8:103143051 | Intergenic region  BAALC |
|  | Translocation | 8:103143146  12:24877293 | BAALC  BCAT1 |
|  | Translocation | 12:11871152  21:34960929 | ETV6  RUNX1 |
|  | Deletion | 12:11883278-24836906 | ETV6-BCAT1 |
| 2 | Translocation | 9:130813896  22:23227330 | ABL1  BCR |
|  | Deletion | 7:50307384-50395933 | IKZF1-IKZF1 |
|  | Deletion | 9:20623612-36945088 | Intergenic region-PAX5 |
| 3 | Translocation | 6:305623  14:106470262 | DUSP22  IGH |
| 4 | Inversion | 16:15721182  16:67089716 | MYH11  CBFB |
| 5 | Deletion | 1:47231900-47313738 | TAL1-STIL |
|  | Deletion | 9:21956507-22072045 | Intergenic region-CDKN2B |

Supplementary Table 3 Comparison between conventional methods and optical genome mapping.

| **Methods** | **Turnaround Time** | **Resolution** | **Strengths** |
| --- | --- | --- | --- |
| Karyotyping/FISH | 14 days-1 month | 5-10Mb | Large structural changes |
| MLPA | 1 week | Targeted 1-2kb | Targeted regions |
| RT-PCR | Hours to 1–2 days | Targeted gene | Targeted gene |
| OGM | 5-7 days | >500 bp | SVs and CNVs comprehensive |
| Short-read WGS | 5-7 days | Single nucleotide | SNV and small indels |

FISH: fluorescence *in situ* hybridization; MLPA: multiplex ligation-dependent probe amplification; RT-PCR: reverse transcription polymerase chain reaction; OGM: Optical genome mapping; SV: structural variation; CNV: copy number variation; WGS: whole genome sequencing; SNV: single nucleotide variation.
